# Gold Nanoparticle-Photosensitizer Conjugate Based Photodynamic Inactivation of Biofilm Producing Cells: Potential for Treatment of *C*. *albicans* Infection in BALB/c Mice

**DOI:** 10.1371/journal.pone.0131684

**Published:** 2015-07-06

**Authors:** Mohd. Asif Sherwani, Saba Tufail, Aijaz Ahmed Khan, Mohammad Owais

**Affiliations:** 1 Interdisciplinary Biotechnology Unit, Aligarh Muslim University, Aligarh, 202002, India; 2 Department of Anatomy, Jawaharlal Nehru Medical College, Faculty of Medicine, Aligarh Muslim University, Aligarh, 202002, India; Louisiana State University, UNITED STATES

## Abstract

**Background:**

Photodynamic therapy (PDT) has been found to be effective in inhibiting biofilm producing organisms. We investigated the photodynamic effect of gold nanoparticle (GNP) conjugated photosensitizers against *Candida albicans* biofilm. We also examined the photodynamic efficacy of photosensitizer (PS) conjugated GNPs (GNP-PS) to treat skin and oral *C*. *albicans* infection in BALB/c mice.

**Methods:**

The biomimetically synthesized GNPs were conjugated to photosensitizers viz. methylene blue (MB) or toluidine blue O (TB). The conjugation of PSs with GNPs was characterized by spectroscopic and microscopic techniques. The efficacy of gold nanoparticle conjugates against *C*. *albicans* biofilm was demonstrated by XTT assay and microscopic studies. The therapeutic efficacy of the combination of the GNP conjugates against cutaneous *C*. *albicans* infection was examined in mouse model by enumerating residual fungal burden and histopathological studies.

**Results:**

The GNP-PS conjugate based PDT was found to effectively kill both *C*. *albicans* planktonic cells and biofilm populating hyphal forms. The mixture of GNPs conjugated to two different PSs significantly depleted the hyphal *C*. *albicans* burden against superficial skin and oral *C*. *albicans* infection in mice.

**Conclusion:**

The GNP-PS conjugate combination exhibits synergism in photodynamic inactivation of *C*. *albicans*. The GNP conjugate based PDT can be employed effectively in treatment of cutaneous *C*. *albicans* infections in model animals. The antibiofilm potential of PDT therapy can also be exploited in depletion of *C*. *albicans* on medical appliances such as implants and catheters etc.

## Introduction

While various species of genus *Candida* often dwell as commensals in healthy individuals, they produce a gamut of serious infections in the immuno-compromised hosts [1–4]. For example, *C*. *albicans* continues to be the major etiological agent [2] for various infectious diseases ranging from superficial skin lesions to severe and invasive systemic candidiasis. Oral candidiasis is one of the various manifestations of *C*. *albicans* superficial infections [5]. In general, fungal infections are difficult to eradicate; the situation gets more aggravated owing to the limited availability of antifungal drugs and the recent trend of development of resistance to most of the existing anti-fungal drugs [6, 7]. Moreover, *C*. *albicans* often adheres to form biofilms on many kinds of surfaces and interfaces (medical implants and catheters) [3]. Long filamentous structures called hyphae are the prominent feature of *C*. *albicans* biofilms [8]. It has been observed that in superficial fungal infections, hyphal filaments penetrate the underlying tissues and help pathogen establishment in the host [6]. Besides other modes, the generation of biofilm offers resistance against antifungal agents and also helps cells to evade host defences [8, 9]. Tackling issues pertaining to treatment of less susceptible pathogenic isolates living in biofilm niche, demand development of alternative treatment modalities that should be effective against microbial biofilms. In this regard, the emerging photodynamic therapy based inactivation of microorganisms offers a potential strategy that holds promises for the treatment of microbial infections in general and fungal infections in particular. Of late various research groups reported potential of PDT in killing of fungal pathogens such as *Candida dubliniensis*, *Aspergillus fumigatus*, *Cryptococcus neoformans*, *Trichophyton rubrum and Saccharomyces cerevisae* etc. [10]. Conceptually, PDT mediated eradication of living cells consists of combination of light source and photosensitizer (PS) mainly [11, 12]. Once exposed to a light of particular wavelength, the photosensitizer gets excited to produce reactive oxygen species (ROS) followed by a series of events which ultimately incur killing of the microbial pathogens [11, 12].

Recently, GNPs owing to their biocompatibility, size and unique surface and optical properties have received significant attention in PDT [13]. Conjugating photosensitizers on the surface of GNPs has become a state-of-the-art approach for efficient *in vivo* activation of photosensitizers to achieve targeted treatment and augmentation of the PDT efficacy [14–16]. In order to develop PDT system in controlling fungal pathogens, methylene blue and toluidine blue O, the two phenothiazine dyes, have been well documented as potential photosensitizers [10, 14, 17–21]. The phenothiazines, in general, target the plasma membrane of fungal cells which gets leaky upon irradiation [21].

Reckoning with the attributes of phenothiazinium dye and gold nanoparticles combination for its potential to kill both susceptible and resistant isolates of *Candida* infections, we evaluated the potential of GNP-PS complex based PDT against *C*. *albicans*. Next, we performed comparative *in vitro* studies of GNP-MB and GNP-TB based inhibition against planktonic *C*. *albicans* cells as well as hyphal forms populating *C*. *albicans* biofilms. Finally, we investigated the efficacy of PDT for the treatment of skin and oral *C*. *albicans* infection in mouse model.

## Materials and Methods

### Chemicals and reagents

All the chemicals and reagents used were of the highest purity available. HAuCl_4_, Methylene Blue, Toluidine Blue O, RPMI 1640, XTT, Chlorpromazine chloride were procured from Sigma Aldrich, USA. Yeast extract, Peptone, dextrose and cotton buds were purchased from Himedia Laboratories Pvt. Ltd. All other reagents used were of analytical grade and procured from local suppliers.

### Preparation of *Aloe vera* leaf extract

Thoroughly washed *Aloe vera* leaves (30 g) were finely cut and boiled in 100 ml sterile distilled water as described earlier [22, 23]. The boiled extract was filtered through Whatman filter and the filtrate was stored at -20°C till further use.

### Synthesis of gold nanoparticles using *Aloe vera* leaf extract

Increasing volumes (1–5 ml of 30% w/v solution) of *Aloe vera* leaf extract prepared following the published procedure [22, 23] were added to 5 ml of 10^−3^ M solution of HAuCl_4_ and the volume was made up to 10 ml by deionized water. The mixture was incubated for a given time period at room temperature (25°C) followed by centrifugation at 20,000 g to pellet the gold nano-particles. The nano-particles were suspended in 1 ml of deionised water and further characterized by various spectrophotometric methods [22–24].

### Characterization of in-house synthesized gold nanoparticles

Spectroscopic and microscopic studies were performed to characterize gold nanoparticles. To obtain UV-visible spectra of in-house prepared gold nanoparticles, they were scanned in range of 300–900 nm using a double beam spectrophotometer. TEM and nanophox particle analyses were used to study the morphological features and size of the particles. The surface properties of gold nanoparticles were analyzed using a transmission electron microscope (1200 EX, JOEL Inc, Peabody, MA) following a method described elsewhere [24]. Samples were prepared by putting a drop of the gold particles on a negative carbon-coated copper grid and dried in air before being transferred to the transmission electron microscope.

### Dyes used as photosensitizers

The two dyes viz. methylene blue and toluidine blue O were used as photosensitizers.

### Conjugation of PSs with gold nanoparticles

Electrostatic interaction mediated conjugation of gold nanoparticles with PSs was achieved following the method described elsewhere [25]. The in-house prepared gold nanoparticles were conjugated with PSs by the reaction of 10 ml colloidal gold solution (pH 9.0) with (equal volume of) 1 mM of MB and TB in PBS. The reaction mixture was incubated for 48 h at room temperature and centrifuged at 20,000 *g* for 15 min at the same temperature. The pellet obtained was dissolved in a minimum amount of 1 mM phosphate buffer (pH 7.4) and lyophilized. The binding of PSs to the nanoparticles was ascertained by analyzing absorption spectra and capturing TEM images of PS conjugated GNPs.

For spectroscopic analysis, a stock solution of 1 mg/ml of various lyophilized preparations was made in PB (pH 7.4). From the stock solutions, an aliquot of 200 μl was picked and made to 1 ml by adding fitting amount of PB so that the final concentration of various formulations was 200 μg/ml. For GNP, MB, TB, GNP-MB and GNP-TB, 200 μl was taken out from the stock solutions (1 mg/ml) of respective preparations and the volume was made to 1 ml by adding PB. However, for the combination of GNP-MB and GNP-TB, an aliquot of 100 μl of each was collected from their respective 1 mg/ml stocks, pooled and the volume was made to 1 ml by adding 800 μl of PB. UV absorbance was measured in the spectral range of 400–700 nm on a double beam Perkin Elmer UV-visible spectrophotometer model λ25 (Boston, MA).

The as-synthesized gold nanoparticle-photosensitizer conjugates were also characterized using TEM. Samples were prepared and observed under the microscope in the same manner as stated earlier for naked GNPs.

### Light exposure

For PDT, a source of light is needed for the production of singlet oxygen *in situ*. We employed an incoherent light source (LumaCare, Newport Beach, CA) delivering full spectrum of visible light (400 nm-800 nm). The instrument provided filter probe of 662 nm for MB and GNP-MB conjugate (exposure time, 20 min) while filter probe of 635 nm was employed for TB and GNP-TB (exposure time, 20 min) mediated PDT against *C*. *albicans*. In case of GNP-MB and GNP-TB combination mediated PDT, filter probes of 662 nm as well as 635 nm were applied in succession for 10 min each. Naked GNPs were subjected to irradiation with filter probe of 532 nm (exposure time, 20 min) because it has been reported that naked GNPs may produce free radicals at this wavelength [13]. The PDT protocol remained same for *in vitro* as well as *in vivo* experiments (except the different filter probes for various PSs) having a total light dose of 21.6 J/cm^2^ with exposure time of 20 min at an irradiance (fluence rate) of 180 W/m^2^ and output power of 120 mW.

### *Candida albicans* culture

All the experiments were conducted using *C*. *albicans* (ATCC 90028) cultured using yeast extract-peptone-dextrose (YPD) medium [24]. Overnight grown culture was centrifuged at 6000 g for 15 min in order to harvest the cells. The harvested cells were washed with PBS and further resuspended in the same buffer to get the OD_570_ of 0.65 of the solution obtained corresponding to a fungal concentration of 10^7^ CFU/ml. We also used *C*. *glabrata* (MTCC 3019) procured from MTCC Chandigarh in our study (see S1 Protocol).

### Microscopic visualization of *C*. *albicans* biofilms

*C*. *albicans* biofilms were grown on sterile plastic coverslips (diameter, 15 mm; Nunc International) placed in six well polystyrene plates (BD Bioscience). After 2 h of incubation of fungal cell suspension (at the density of 10^7^ cells) onto the coverslips at 37°C, coverslips were washed three times to remove un-adhered cells. Further, the plastic coverslips were incubated with 400 μl of RPMI media for another 24 h at 37°C to allow biofilm development. Thereafter, coverslips were treated with 200 μg/ml (50 μl) of GNP, MB, TB, GNP-MB, GNP-TB and GNP-MB+GNP-TB for 30 min under dark conditions. An aliquot of 10 μl was picked from 1 mg/ ml stock solutions of various preparations and made to 50 μl by adding fitting amount of PB. For GNP-MB and GNP-TB combination, aliquots of 5 μl were picked from their respective stock solutions, pooled and made to 50 μl by adding PB. After treatment with various formulations, the biofilm was subsequently exposed to the light source of respective wavelengths for 20 min according to the PS used to execute photodynamic killing of the fungal cells. In case of GNP-MB and GNP-TB combination, filter probes of 662 nm as well as 635 nm were applied in succession. Finally, coverslips were fixed in 2% paraformaldehyde followed by washing and visualized under fluorescence microscope.

For scanning electron microscopy (SEM) of *C*. *albicans* biofilms, a published protocol was followed [26]. Briefly, biofilm formation was initiated on sterile plastic coverslip discs in 6 well cell culture plates by dispensing a standardized cell suspension (2 ml of a suspension containing 1 x 10^6^ cells/ml in RPMI 1640) onto appropriate discs at 37°C for 2 h. Subsequently, cells were washed to remove non-adhered cells for biofilm formation. Coverslip discs were incubated for another 24 h in 400 μl of RPMI media. Thereafter, coverslips were treated with 200 μg/ml (50 μl) of various formulations viz. GNPs, MB, TB, GNP-MB, GNP-TB and GNP-MB+GNP-TB (as detailed above) for 30 min followed by exposure to light of respective wavelengths for 20 min. The discs were removed and washed three times in sterile PBS. The biofilms were placed in fixative (4% [vol/vol] formaldehyde and 1% [vol/vol] glutaraldehyde in PBS) overnight. The samples were rinsed twice (3 min each) in 0.1 M phosphate buffer and then placed in 1% osmium tetraoxide for 30 min. The samples were subsequently dehydrated in a series of ethanol washes (70% ethanol for 10 min, 95% ethanol for 10 min, 100% ethanol for 20 min), and finally air dried in a desiccator. The specimens were then coated with 40% gold–60% palladium and observed with a scanning electron microscope (JEOL) in high-vacuum mode at 15 kV.

### Biofilm quantitation by XTT assay

#### Quantitation of mature biofilms

To form *C*. *albicans* biofilm, ninety six well sterile polystyrene plates were inoculated with 100 μl (10^7^ cells) of the standardised cell suspension and left for 2 h at 37°C under shaking conditions to induce cell adhesion [9]. The plates were washed three times with PBS in order to remove unattached planktonic cells. Finally, 100 μl of RPMI media was dispensed to each well and plates were incubated for 24 h at 37°C for the development of biofilms. Biofilm formation was quantitated using XTT reduction assay method [27]. The wells containing mature biofilms developed after 24 h were washed with PBS to remove non-adhered cells. Thereafter, mature biofilms were treated with various formulations viz. GNPs, MB, TB, GNP-MB, GNP-TB and GNP-MB+GNP-TB for 30 min and later exposed to light of respective wavelengths for 20 min. GNP and GNP-PS formulations were lyophilized and a stock solution of 1 mg/ml of various formulations was made in PB. Stock solutions of 1 mg/ml in PB for MB as well as TB were made. An aliquot of 10 μl was picked from the stock solutions of GNP, MB, TB, GNP-MB and GNP-TB and was made to 100 μl by adding 90 μl of RPMI media so that its concentration became 100 μg/ml (10 μg/100 μl). For the combination of GNP-MB and GNP-TB, 5 μl of each was picked from their respective stock solutions, pooled and made to 100 μl by adding fitting amount of RPMI media (90 μl). Then the aliquots of various formulations with concentration of 10 μg/100 μl (100 μg/ml) were dispensed into the wells and serially diluted. After treatment and light exposure, the plates were incubated for 5 h in 100 μl of XTT menadione solution in dark, at 37°C using rotator incubator (100 rpm). Briefly, XTT solution was prepared by mixing 1 mg/ml XTT salt in PBS and stored at -20°C. Before commencement of incubation, the menadione solution prepared in acetone was added to XTT solution to achieve a final XTT concentration of 4 μM. The color formation by water soluble formazan product was measured at 450 nm using a microplate reader (BioRad, USA). Wells without biofilms served as a blank.

#### Quantitation of developing biofilms

*C*. *albicans* biofilms were grown in ninety six-well sterile polystyrene plates. After 2 h of incubation of fungal cell suspension (at the density of 10^7^ cells) in the wells at 37°C, plates were washed three times to remove un-adhered cells. Further, the plates were incubated with 100 μl/well of RPMI media for another 4 h at 37°C to allow biofilm development. After 4 h incubation, when biofilms were still developing, biofilms were given dark exposure of various formulations viz. GNPs, MB, TB, GNP-MB, GNP-TB and GNP-MB+GNP-TB for 30 min followed by irridiating light of respective wavelengths for 20 min (the concentrations of various formulations were same as stated for mature biofilms). After treatment and light exposure, the plates were incubated for another 20 h at 37°C. Finally, the plates were incubated for 5 h in 100 μl of XTT menadione solution in dark, at 37°C using rotator incubator (100 rpm). The color formation by water soluble formazan product was measured at 450 nm using a microplate reader (BioRad, USA). Wells without biofilms served as a blank.

### Gene expression in *C*. *albicans* biofilm

Quantitative real-time reverse transcription-PCR (RT-PCR) was used to compare mRNA abundances of the genes of interest. Details of the procedure can be found in S2 Protocol.

### Photodynamic inactivation of *C*. *albicans* cells *in vitro*

To evaluate the effect of photodynamic therapy on planktonic cells, the *C*. *albicans* suspension at the density of 10^7^ CFU/ml in PBS was dispensed into a six well culture plate and incubated with 200 μg/ml (50 μl) of various viz. naked GNP, MB, TB, GNP-MB, GNP-TB and GNP-MB+GNP-TB formulations (as detailed in ‘microscopic visualization of *C*. *albicans* biofilm’) for 30 min at room temperature in the dark, the fungal cell post GNP-PS exposure, were irradiated with the light source of respective filter probes for 20 min. During illumination, the lids of the culture plates were removed. Aliquots of 50 μl of the fungal suspension were withdrawn and plated onto the YPD agar plates using the published method [7, 28]. Photodynamic inactivation of *C*. *glabrata* was also performed (see S1 Protocol).

### Animals

Inbred female BALB/c mice (6–8 weeks old, 20 ± 2 g) were obtained from the Institute’s Animal House Facility of Interdisciplinary Biotechnology Unit, Aligarh Muslim University. Mice were quarantined for two weeks under standard husbandry conditions at room temperature (21°C ± 4°C), relative humidity (65% ± 10%) and 12-hour light/dark cycle. The animals were housed in polypropylene cages on wood powder beddings and allowed free access to dry pellet feed diet (Ashirwad, Chandigarh, India) and water *ad libitum* under strict hygienic conditions. **Ethics statement**. All animal experiments were performed in strict accordance with the National Regulatory Guidelines issued by the Committee for the Purpose of Control and Supervision of Experiments on Animals, Govt. of India (CPCSEA). Our approval ID was 332/CPCSEA, Ministry of Environment and Forest, Government of India. All the procedures used for the animal experiments were reviewed and approved by the Institutional Animal Ethics Committee of the Interdisciplinary Biotechnology Unit, Aligarh Muslim University, Aligarh, India. Animals were anesthetized with ketamine (100 mg/kg body weight) in combination with xylazine (5 mg/kg body weight) prior to dorsal shavings, tongue infection and sacrifice. In all experimental procedures, efforts were made to minimize pain and suffering.

### PDT efficacy against intracellular *C*. *albicans* in macrophages

Peritoneal macrophages isolated following the published protocol [29] were plated at the density of 4 x 10^5^ cells /well. After 2 h adherence, inocula of 3 x 10^5^ CFU of *C*. *albicans* were added to the monolayer in each of the wells in 24 well plates. After 30 min, the non-ingested yeast was removed by washing the monolayer three times with the DMEM medium. The macrophages were re-incubated for 24 h with 200 μg/ml (50 μl) of various formulations viz. GNP, MB, TB, GNP-MB, GNP-TB and GNP-MB+GNP-TB (as detailed in ‘microscopic visualization of *C*. *albicans* biofilm’) and further, light of respective wavelengths was irradiated for 20 min. The number of *C*. *albicans* inside the infected macrophages was calculated by lysing the macrophages with Triton X-100 (0.2%) and sub-culturing in YPD plates and compared with an untreated control.

### Topical skin *C*. *albicans* infection in experimental mice

The mice were anesthetized by intraperitoneal (i.p.) injection of a ketamine-xylazine cocktail and then shaved on the dorsal surfaces. Mouse skin was scraped with sterile scalpel blades until a reddened area appeared (just short of bleeding). Each wound measured approximately 1.2 cm by 1.2 cm. With the help of a pipette tip, the surface of each wound was inoculated with 40 μl of the prepared culture suspension corresponding to 10^7^ CFU of *C*. *albicans*, which was smeared onto the wound surface with an inoculating loop. Colonies were allowed to grow for 24 h at 25°C.

### PDT against mouse skin *C*. *albicans* infection

The potential of PDT against skin *C*. *albicans* infection was assessed in BALB/c mice. PDT was initiated after 24 h of exposure of animals to fungal inoculum to establish the treatment efficacy of PDT. Before light irradiation, 50 μl (200 μg/ml) of GNP-PS solution was smeared onto each wound and kept for 30 min in the dark. Mice were given a total light exposure of up to 21.6 J/cm^2^ for 20 min. The light was delivered at an irradiance of 180 W/m^2^ and the output power was 120 mW.

### Induction of oral candidiasis in mice and PDT therapy

To study the photodynamic effect of various formulations on oral candidiasis, mice were immunosuppressed by following the protocol published elsewhere. Briefly, a single intraperitoneal injection of cyclophosphamide (250 mg/kg) was given to each mouse to induce neutropenia that persisted temporarily for five to seven days after cyclophosphamide treatment. Small cotton buds soaked in a *C*. *albicans* cell suspension (2.5 x 10^7^ cells/ml), were put in oral cavity of the anesthetised mice to induce oral infections [5]. Post day 7 to exposure with fungal challenged animals was treated with PDT. GNP-PS solution (50 μl, 200 μg/ml) was smeared onto the infected tongues followed by 20 min exposure to light source of respective wavelength. Mice were given a total light exposure of up to 21.6 J/cm^2^. The light was delivered at an irradiance of 180 W/m^2^ and the output power was 120 mW.

### Histopathological studies

Animals were sacrificed and their excised skin tissues and tongues (in case of oral candidiasis) were immersion fixed in Karnovsky's fixative agent. Next, the tissue blocks of 3 × 6 × 5 mm^3^ dimensions were processed for paraffin embedding. Ten micron and five micron thick sections of fixed skin tissue and tongues respectively were cut with rotary microtome from paraffin blocks and stained with periodic acid-Schiff stain for histopathological examination and fungal detection (PAS) [7]. Observations were made under light microscope (Olympus-BX 40-Japan), representative photomicrographs with final magnification of X 400 were used for comparative study.

### Statistical analysis

Statistically groups treated with various formulations viz. GNPs, MB, TB, GNP-MB, GNP-TB and GNP-MB+GNP-TB were compared with untreated control using ANOVA (Analysis of Variance) with the Holm—Sidak test. *P* values <0.05 were considered statistically significant.

## Results

### Characterization of gold nanoparticles

**Fig 1:**
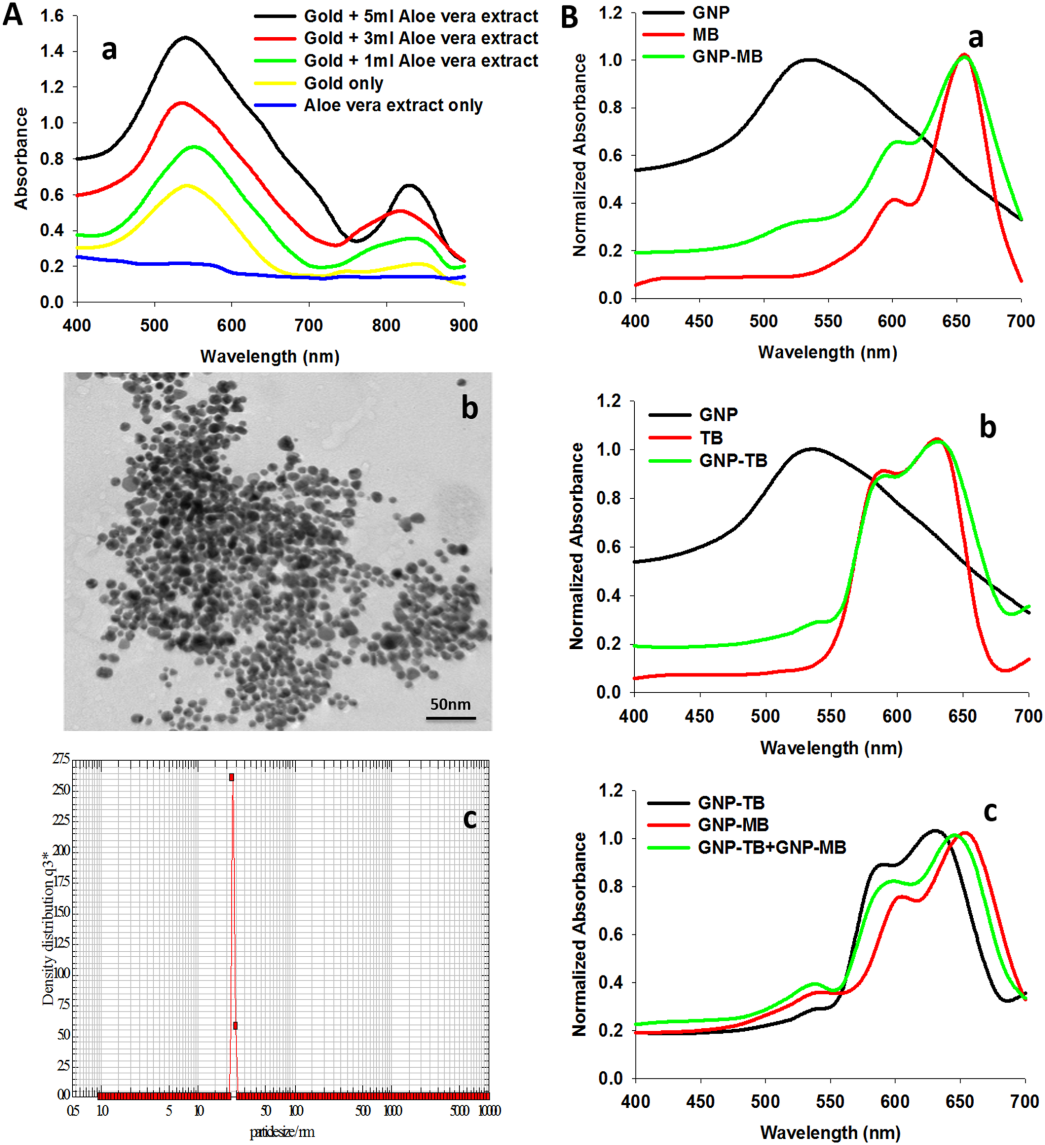
Characterization of in-house synthesized GNPs and PS conjugation onto their surface. **(A)** Characterization of in-house synthesized gold nanoparticles. (a) Ultraviolet-visible- near infrared spectra of gold nanoparticles synthesized by exposing various amounts of *Aloe vera* extract to a fixed volume of HAuCl4 solution (10^−3^ M), keeping the final volume of reaction mixture 10 ml that was made up for each sample by increment in *Aloe vera* extract content for 24 h. Absorbance is seen to be increasing exhibiting two major peaks, a larger one at 540 nm, the characteristic peak of GNPs. (b) Representative TEM image of gold nanoparticles synthesized using *Aloe vera* leaf extract exhibiting a large population of spherical gold nanoparticles of approx. 10 nm size. (c) Nanophox particle analysis of synthesized gold nanoparticles. **(B)** Ultraviolet-visible absorption spectra of gold nanoparticle-photosensitizer conjugate normalized to the maximum absorbance intensity. GNP has signature spectra at 540 nm due to surface plasmon resonance. MB shows absorption peaks at 662 nm and 613 nm. Conjugation of MB to GNP led to appearance of a small peak at 540 nm, intrinsic feature of GNP, which was absent in pure MB spectrum (a). TB spectrum enumerates peaks at 625 nm and 585 nm. GNP-TB conjugate is observed to exhibit an additional shoulder at 540 nm indicating conjugation of GNP to TB (b). Spectrum obtained for the mixture of GNP-MB and GNP-TB (c).

The gold nanoparticle synthesis was established by characteristic UV-visible spectra. As shown in Fig 1Aa, increase in the amount of plant extract results in an increase in the absorbance at 540 nm [24]. Further, microscopic analysis also ascertained GNPs synthesis. TEM analysis was performed with the nanogold solution harnessed after 24 h of incubation of HAuCl_4_ with 5 ml of *Aloe vera* leaf extract. Fig 1Ab shows representative TEM image displaying majorly spheroidal gold nanoparticles. Particles are seen to be in the size range of 10–20 nm. The size of the gold nanoparticle was also confirmed by nanophox analysis (Fig 1Ac).

### Conjugation of gold nanoparticles with photosensitizers

**Fig 2:**
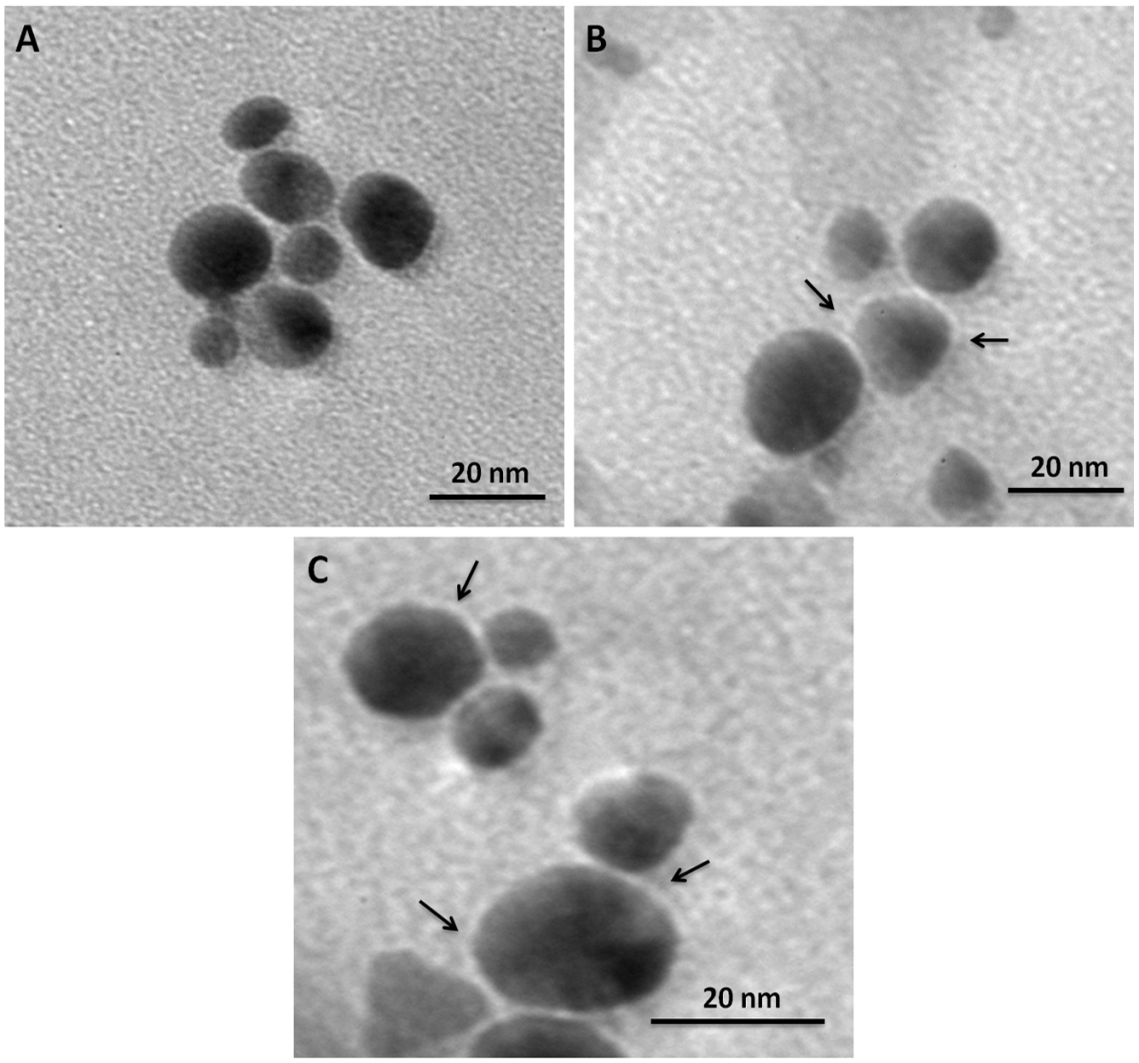
Transmission electron microscopic images of GNP, GNP-TB and GNP—MB. **(A)** Biomimetically synthesized naked spherical colloidal GNPs **(B)** MB-conjugated GNPs (C) TB-conjugated GNPs. Coating of PSs around GNPs is indicated by black arrows.

Dispersion of colloidal gold nanoparticles in a basic solution (pH 9.0) helped them acquire negative surface charge. The positively charged photosensitizers were allowed to interact electrostatically with GNPs. The conjugation was ascertained by visible spectroscopy and TEM analysis. Fig 1B depicts the UV-visible absorbance spectra of PS conjugated GNPs normalized to the maximum absorbance intensity (the original or un-normalized absorption spectra are shown in S1 Fig). As exhibited in Fig 1B, GNP-MB and GNP-TB conjugates show an additional small shoulder at 540 nm, a characteristic of GNPs which is absent in either unconjugated MB or TB spectrum. The appearance of this additional peak at 540 nm in conjugates suggests conjugation of MB and TB to the GNPs. Furthermore, TEM images also highlight the conjugation of MB and TB to GNP surface (Fig 2). A layer of MB and TB seems to be coated on the surface of dye conjugated GNPs as shown by the arrow heads.

### PDT induced regression in *C*. *albicans* biofilm

**Fig 3:**
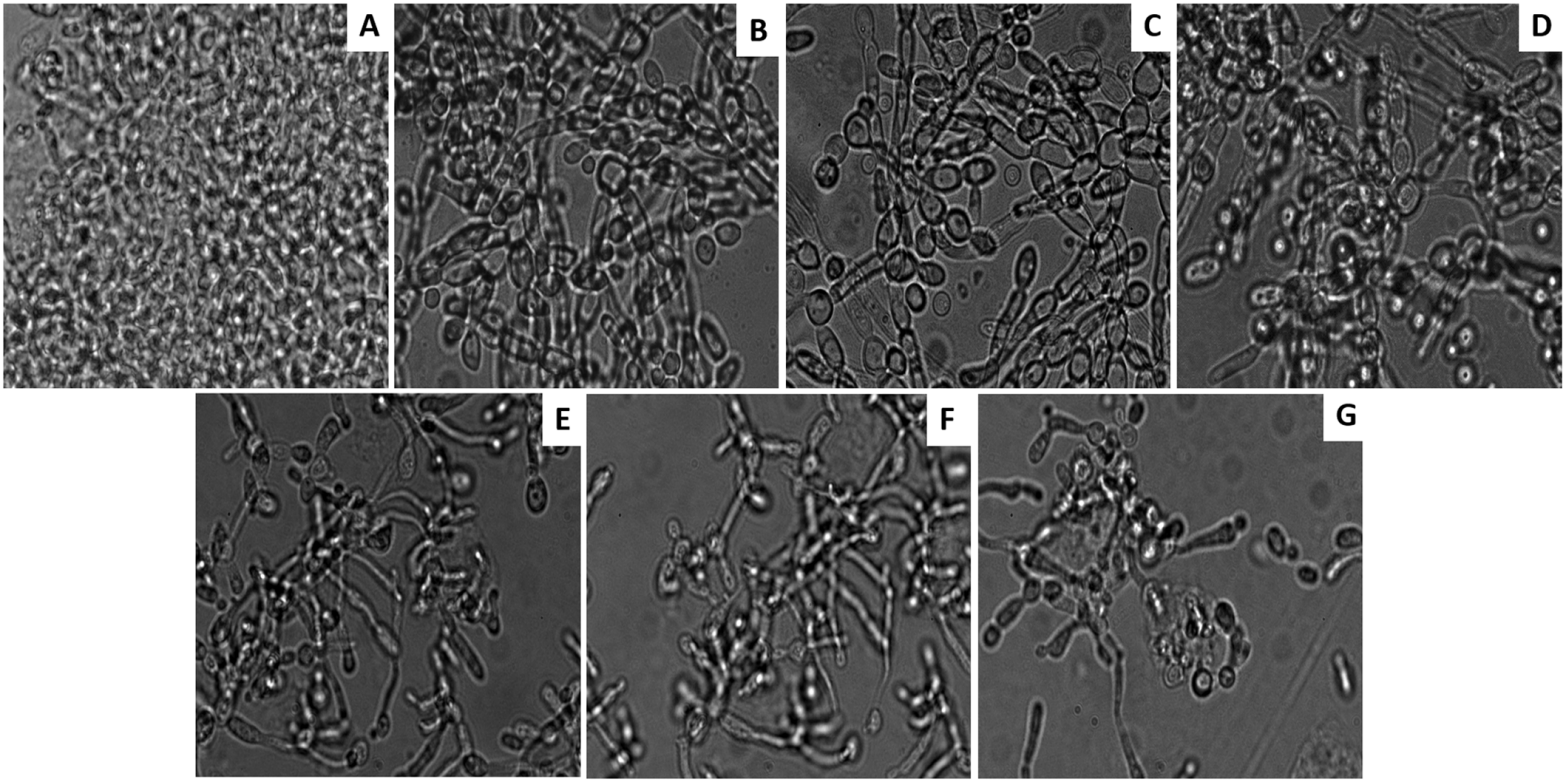
Microscopic images revealing photodynamic inhibition of *C*. *albicans* biofilm grown on plastic coverslips. **(A)** Untreated control image of the *C*. *albicans* biofilm **(B)** Biofilm treated with naked GNPs **(C)** Biofilm exposed to MB **(D)** Biofilm after treatment with TB **(E)** GNP-MB **(F)** GNP-TB treated biofilms **(G)** GNP-MB and GNP-TB combination exposed *C*. *albicans* biofilm.

**Fig 4:**
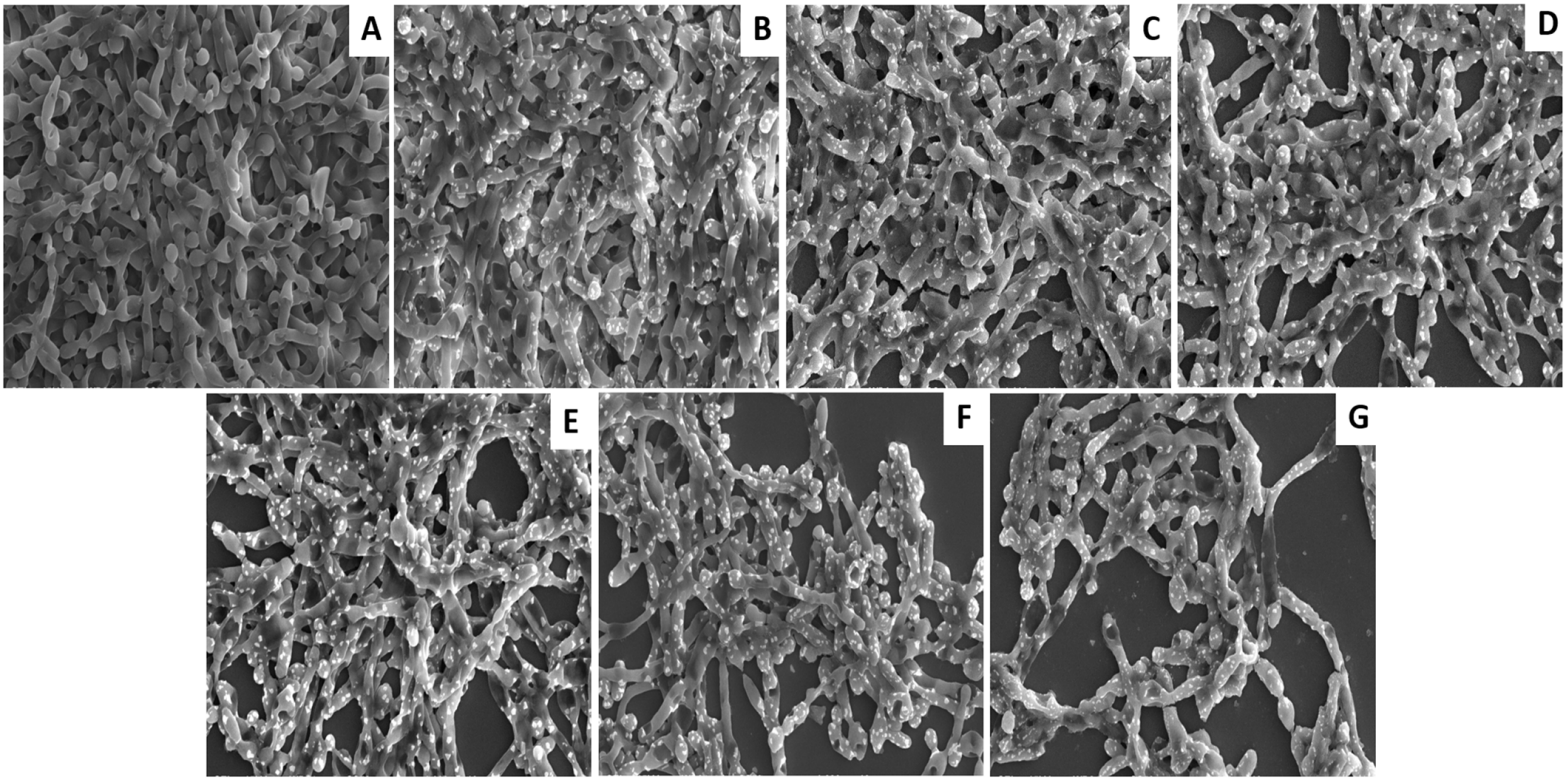
SEM micrographs showing marked inhibition of *C*. *albicans* biofilm by GNP-PS (GNP-MB and GNP-TB) combination mediated PDT. **(A)** Untreated control image of the *C*. *albicans* biofilm **(B)** Naked GNPs treated biofilm **(C)** *C*. *albicans* biofilm treated with unconjugated MB **(D)** Biofilm exposed to TB **(E)** Biofilm treated with GNP-MB and **(F)** GNP-TB treated biofilm **(G)** GNP-MB and GNP-TB combination mediated reduction in biofilm.

Both naked as well as PS conjugated GNPs were evaluated for their potential to inactivate *C*. *albicans* biofilms grown on plastic coverslips. Figs 3 and 4 display a marked inactivation of *C*. *albicans* biofilm upon its sensitization with either GNP-MB or GNP-TB when compared to control. Interestingly, a more pronounced reduction in the biofilm was observed upon its exposure to mixture of GNP-MB and GNP-TB than for the conjugates taken solitarily (Figs 3G and 4G). Although GNP, MB and TB exhibited slight reduction in biofilm as compared to untreated control but the results are much feeble than observed for the conjugates.

**Fig 5:**
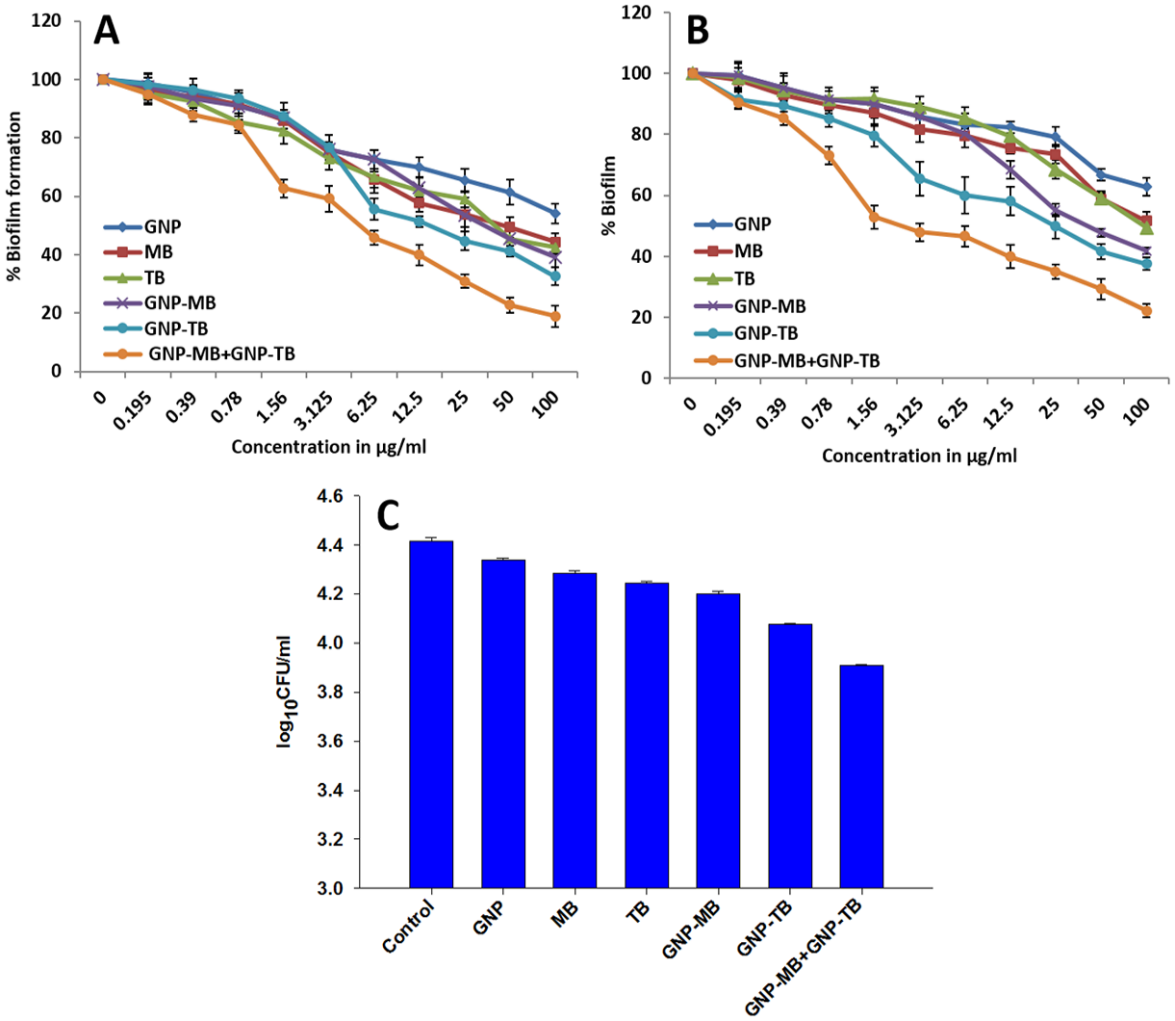
Photodynamic effect of GNP-MB and GNP-TB combination on *C*. *albicans* biofilm and planktonic cells. Effect of GNP-MB and GNP-TB alone and in combination against biofilm development **(A)** and mature biofilm **(B)**. Growth percentage was analyzed by comparing relative metabolic activity (RMA) obtained through XTT assay taking untreated control as 100%. Photodynamic inactivation of planktonic *C*. *albicans in vitro* **(C).** *C*. *albicans* yeast cells incubated with GNP-PS complexes, after being irradiated with respective light source were picked (50 μl) and plated onto YPD agar plates for counting CFU. The data represent the mean of three determinants ± SD (Standard Deviation) and are representative of three different experiments (i.e, the experiment was done in triplicate) with similar observations. Statistically groups were compared with control using ANOVA (Analysis of Variance) with the Holm—Sidak test. *P* values <0.05 were considered statistically significant. GNP-MB+GNP-TB vs Control, *P*<0.005.

The XTT assay was performed quantitatively to assess potential of PDT against developing biofilms and mature biofilms (Fig 5A and 5B respectively). Although considerable inhibition of biofilm growth and depletion of mature biofilm was observed upon exposure with either of the PS conjugates, however, therapy employing both GNP-MB and GNP-TB combination simultaneously produced a marked synergistic inhibition of biofilm development as well as marked depletion of mature biofilm. For GNP-MB and GNP-TB conjugates, approx. 40–50% viable biofilm was observed both in the immature as well as fully developed film but the mixture of the two conjugates rendered a significant inhibition and only 18% and 20% viable biofilm could be quantified for immature and mature biofilm respectively at a conc. of 100 μg/ml for the mixture of conjugates.

### Photodynamic inactivation of *C*. *albicans in vitro*

To quantitate photodynamic inactivation of *C*. *albicans* cells YPD agar plates were counted for a comparison of CFU for various conjugates. Fig 5C depicts that exposure of 21.6 J/cm^2^ light dose led to significant inactivation of fungal cells as compared to control when the *C*. *albicans* cells were incubated with the mixture of gold nanoparticle-PS conjugates (GNP-MB+GNP-TB) (*P*<0.005). Both GNP-MB and GNP-TB also induced significant reduction in CFU as compared to control, but highest reduction was observed for GNP-MB and GNP-TB combination (Fig 5C).

### Uptake of gold nanoparticle-photosensitizer complex by macrophages

**Fig 6:**
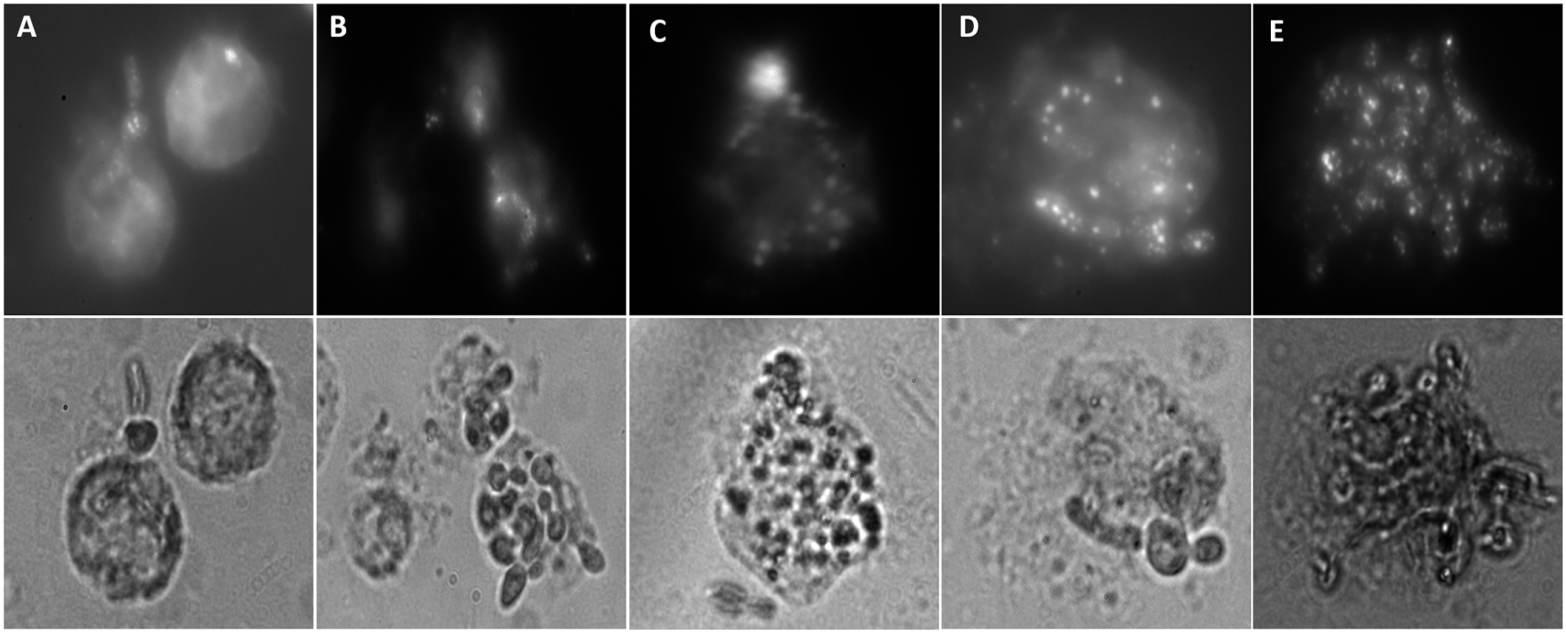
Uptake of GNP-PS conjugates by activated macrophages. **(A)** Macrophages exposed to MB **(B)** TB treated macrophages **(C)** GNP-MB conjugate entrapped by macrophages **(D)** GNP-TB complex engulfed by macrophages **(E)** Macrophages are seen with GNP-MB and GNP-TB entrapped within. Upper panel shows fluorescent images and lower panel exhibits the corresponding brightfield images.

Since yeast form of *C*. *albicans* can intrude the macrophages [30], it can be speculated that uptake of GNP-PS conjugates by macrophages would render close proximity of PS with intracellular *C*. *albicans*. Upon subsequent light exposure, free radicals generated by PSs would readily act upon the *C*. *albicans* cells present in the vicinity ensuing in effective killing of the pathogens. Consequently, we investigated the cellular uptake of GNP-PS complex in macrophages isolated from the peritoneal cavity of thioglycolate primed BALB/c mice. The isolated macrophages were incubated with PS conjugated GNPs. Endocytosis of the GNP-PS complexes by the macrophages appears in the form of punctate fluorescence (Fig 6). The punctuate fluorescence intensity in the macrophages incubated with the mixture of GNP-MB and GNP-TB is significantly higher when compared to that incubated with either of the conjugates alone. The macrophages exhibited very feeble punctuate fluorescence for the unconjugated MB and TB possibly because of their non-particulate nature (Fig 6).

### PS induced *in vitro* killing of intracellular *C*. *albicans* within macrophages

**Fig 7:**
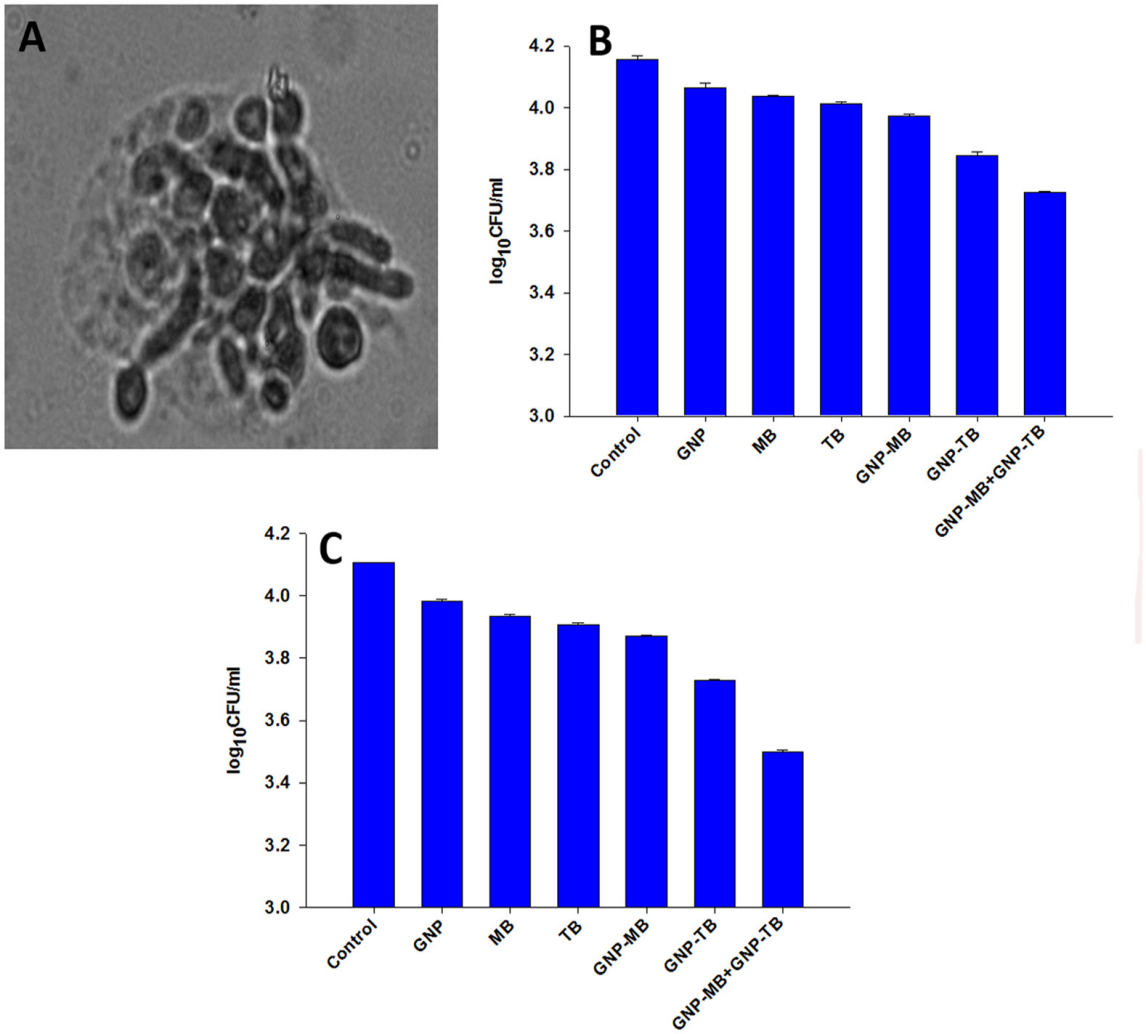
GNP-PS conjugate mediated killing of *C*. *albicans* inside macrophages and reduction of fungal burden in skin *C*. *albicans* infection. **(A)** *C*. *albicans* yeast cells and hyphal filaments are seen to be harbouring the intracellular niche of macrophage. **(B)** GNP-MB and GNP-TB mixture is exhibited to be the best amongst various formulations taken in inhibiting *C*. *albicans* residing within the macrophages. **(C)** PDT employing GNP-MB and GNP-TB conjugates result in reduced fungal burden in skin *C*. *albicans* infection. After a total light exposure of 21.6 J/cm^2^, GNP-PS complex treated wounds were excised aseptically. The homogenate was plated onto YPD agar plates to count the CFU. The combination of GNP-MB and GNP-TB, in a manner similar to *in vitro* results, depleted the fungal burden in the skin most efficiently when compared to other formulations. Data are representative of three independent experiments ± SD values. *P* values<0.05 were considered significant. GNP-MB+GNP-TB vs Control, *P*<0.005.

Before proceeding to the *in vivo* studies, we ascertained the photodynamic inactivation of *C*. *albicans* inside the macrophages *in vitro*. Macrophages loaded with *C*. *albicans* and GNP-PS conjugates were irradiated with respective filter probes. As shown in Fig 7A, macrophages endocytose GNP-PS conjugates causing reduction in fungal cell population. GNP-MB and GNP-TB combination rendered a significant decrease in CFU when compared to control (*P*<0.005). On the other hand, GNP-MB and GNP-TB when used alone could bring about a relatively lower inhibition as compared to the combination of conjugates (Fig 7B).

### Efficacy of PDT in treatment of topical *C*. *albicans* skin infection in mice

Once the efficacy of PDT employing GNP-PS conjugates against *C*. *albicans* (both planktonic and hyphal forms in biofilm) was established *in vitro*, we set about to evaluate the efficacy of PDT for treating cutaneous infections. The cutaneous fungal infections are characterized by *C*. *albicans* occupying the cytosolic niche of epidermal macrophages. As shown in Fig 7C, GNP-MB+GNP-TB exhibited almost 50% reduction in *C*. *albicans* CFU when compared to untreated control (*P*<0.005) indicating the synergistic efficacy of GNP-MB+GNP-TB in treating skin *C*. *albicans* infections. In compliance with *in vitro* killing efficacy, both GNP-MB and GNP-TB solitary treatment was found to be less efficacious when compared to their combination.

### Potential of PDT in treating skin and oral candidiasis as revealed by histopathological analysis

**Fig 8:**
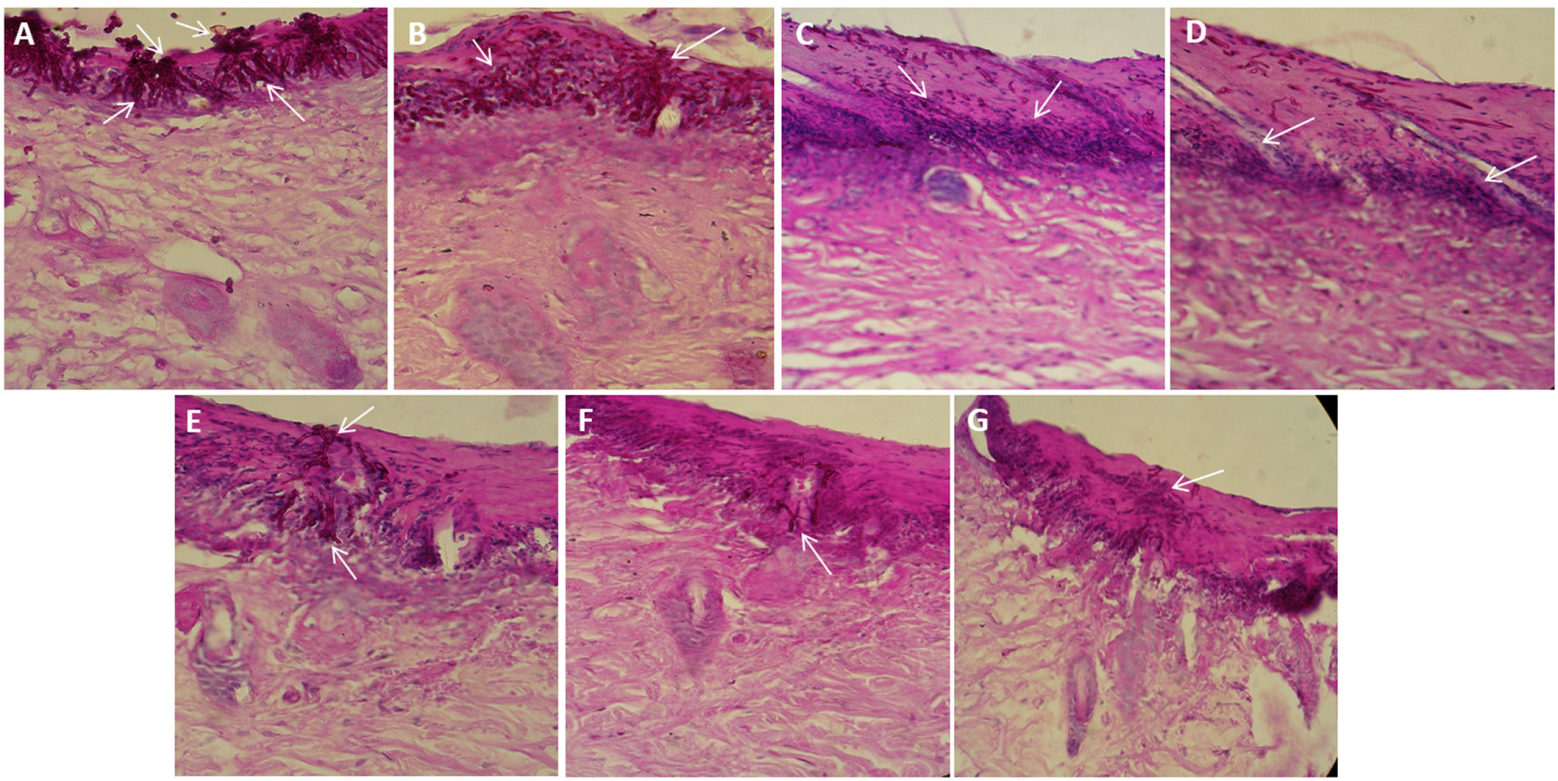
Representative images of mouse skin infected with *C*. *albicans* and stained with periodic acid—Schiff; X400. **(A)** Untreated control, showing exuberant growth **(B)** Naked GNPs, showing only mild response to treatment **(C)** MB treated group and **(D)** TB exposed group, exhibiting better response to treatment than GNPs **(E)** GNP-MB group and **(F)** GNP-TB group, showing inhibition on the growth and hyphal population is also observed to be highly decreased in comparison to control. **(G)** Combination of both GNP-MB and GNP-TB showing significant inhibition on the growth. The arrows indicate the population of *C*. *albicans* yeast cells and hyphae penetrating the skin tissue.

For histopathological studies, skin as well as tongue specimens obtained from infected and PDT treated mice were fixed and stained as described in materials section for analyzing and comparing the efficacy of PDT employing GNP-MB and GNP-TB either alone or in combination for treatment of superficial skin as well as oral *C*. *albicans* infection. Fig 8 shows the representative infected skin and tongue sections respectively stained with PAS. As shown in Fig 8, the control image (untreated skin wound) demonstrates the presence of innumerable *C*. *albicans* yeast cells and tissue penetrating hyphal filaments whereas the infected wounds treated with GNP-PS mediated PDT exhibited pronounced reduction in the yeast cells and hyphal filaments. Yeast cells can be observed on the cell surface while hyphal filaments are seen to be penetrating the underlying tissue and branching in multi-directions. Naked GNP, MB, TB, GNP-MB and GNP-TB exposed wounds were found to harbour lower yeast cells and hyphal filaments than control yet the reduction is not as marked as seen in the wounds treated with the GNP-MB and GNP-TB combination (Fig 8). Hence, the potential of GNP-PS conjugates was also successfully translated in *in vivo* conditions.

**Fig 9:**
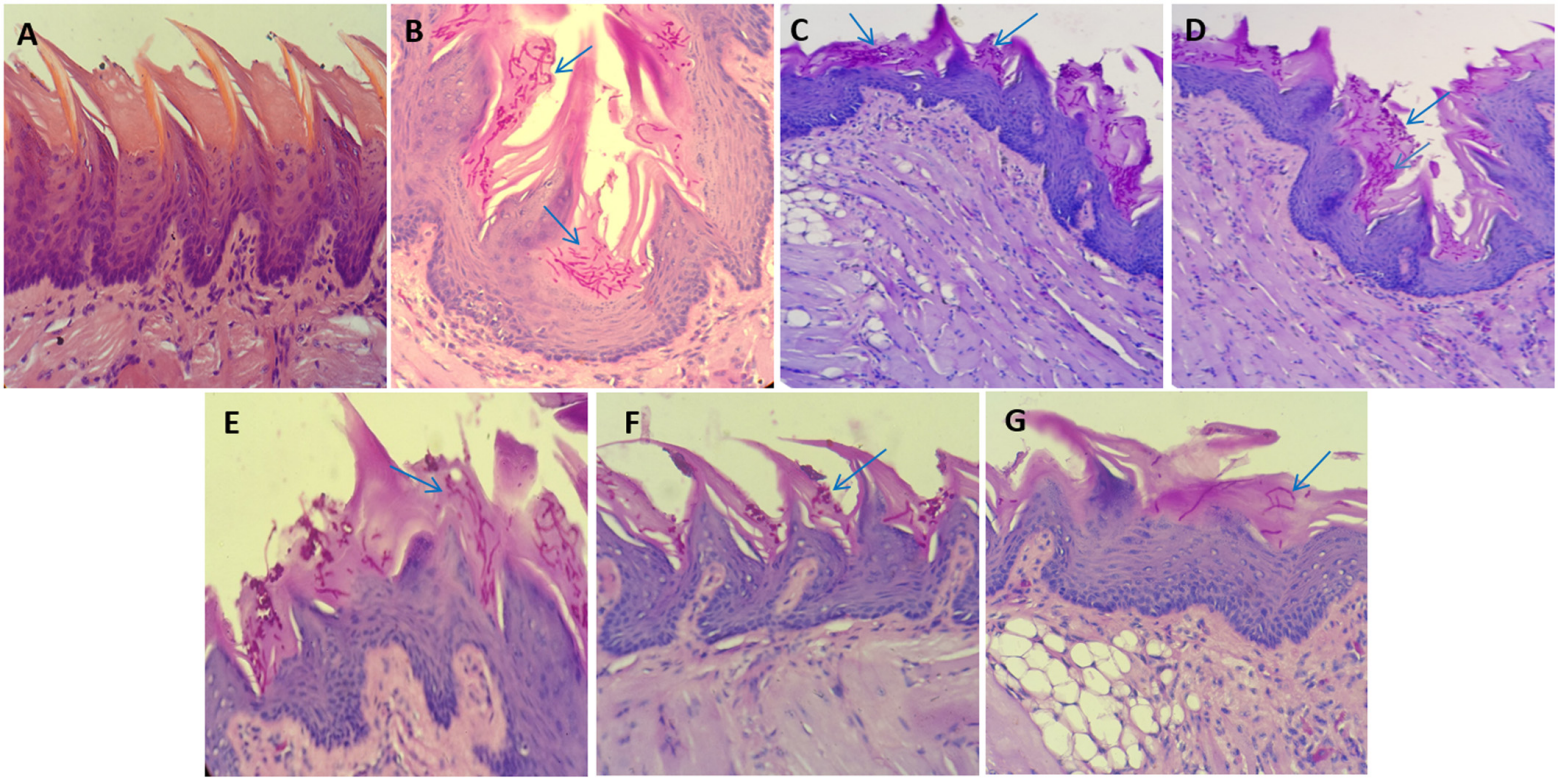
Sagittal cut of the tongue dorsum of mouse oral candidiasis model. Representative images of mouse tongue dorsum infected with *C*. *albicans* and stained with periodic acid—Schiff; X400. **(A)** Healthy control, showing numerous elongated papillae. **(B)** Untreated control showing numerous yeast cells and hyphal filaments inhabiting the tongue papillae **(C)** MB treated group and **(D)** TB treated group showing mild response to treatment **(E)** Mice tongue treated with GNP-MB and **(F)** GNP-TB treated group, showing comparatively better inhibition of hyphal population than control or unconjugated PS treated groups **(G)** Combination of both GNP-MB and GNP-TB showing marked inhibition on the growth of yeast cells as well as hyphae. The population of *C*. *albicans* yeast cells and hyphae penetrating the tongue dorsum is indicated by the arrows.

GNP-MB and GNP-TB combination was also found to be effective in treating oral candidiasis as enumerated by Fig 9. Numerous yeast cells and hyphal filaments are observed harboring the papillae as well as papillary spaces in the untreated control. Upon treatment with various GNP-PS conjugates, a reduction in yeast cells and hyphal filaments is seen although the best result is obtained for GNP-MB and GNP-TB combination where a marked reduction in fungal population is observed with negligible yeast cells and only few hyphal filaments sparsely scattered in the papillae after treatment. MB and TB when given alone also exhibit reduction in fungal population (yeasts and hyphae) as compared to untreated control but the results are not as good as those observed for the conjugates (Fig 9).

## Discussion

*C*. *albicans* biofilm, like many other microorganisms, arise from micro-colonies embedded in an extracellular matrix [8, 31–34]. The prominence of hyphal filaments is a hallmark of *C*. *albicans* biofilm maturation [8]. Besides assisting host defence evasion, hyphal transformants are lethal to macrophages and endothelial cells [35, 36]. The increasing incidences of fungal infections combined with generation of resistance to the limited available drugs warrant development of alternative treatment modalities.

While both chemo-therapy and photodynamic therapy were perceived with equal fervour and promise, the former has enjoyed an extra edge leading to undermining the utility of PDT. Recently, it has been reported that PS conjugated gold nanoparticle based PDT is more effective against microbial elimination when compared to the free dye [14, 37–39]. Reckoning with this perspective, we evaluated efficacy of the combination of gold nanoparticle conjugated MB and TB against *C*. *albicans* both *in vitro* and *in vivo* system. The data obtained from the present study demonstrated that PDT, employing a combination of GNP-MB and GNP-TB, can significantly inhibit the planktonic (yeast) as well as hyphae populating biofilm of *C*. *albicans in vitro*. The cutaneous fungal infections are mostly characterized by the hyphal filaments penetrating the tissues in various directions. Keeping this fact into consideration, we evaluated PDT efficacy in treatment of topical skin and oral *C*. *albicans* infection. The treatment studies showed that GNP-MB and GNP-TB combination can markedly reduce fungal burden (both yeast cells and hyphal filaments) in topical skin wounds and tongues infected with *C*. *albicans* in BALB/c mice.

Biomimetically synthesized gold nanoparticles were characterized on the basis of UV-Vis absorption spectroscopy and transmission electron microscopy. Gold nanoparticles in the size range of 10–20 nm are seen majorly as spheroids in the TEM image (Fig 1A). The conjugation of MB and TB to GNP surface was also ascertained by UV-Vis spectroscopy and TEM analysis. As observed in Fig 1B, the spectra for GNP-MB and GNP-TB are quite different from spectra of naked GNPs and PSs alone. GNP-PS spectrum exhibit a shoulder at 540 nm, the signature peak of GNP, which is absent in unconjugated PSs. The hybrid spectra indicate the conjugation of both phenothiazine dyes to GNPs. This was further confirmed by TEM analysis highlighting adsorption of PSs around naked GNPs (Fig 2).

GNP-PS conjugates either alone or in combination were evaluated for anti-*C*. *albicans* biofilms activity as well. First, we examined inhibition of *C*. *albicans* biofilms. As seen in microscopic images (Figs 3 and 4), a significant reduction in *C*. *albicans* biofilm was observed upon treatment with GNP-MB and GNP-TB combination. Both GNP-MB and GNP-TB alone treatment could bring significantly less *C*. *albicans* inhibition when compared to combination therapy. The efficacy of PDT on Candida biofilm was evaluated quantitatively employing XTT reduction assay (Fig 5A and 5B). Inhibition to yeast hypha transition and also on the biofilm was found to be concentration dependent. Interestingly, the strategy seems to work equally well on established mature biofilm as well. XTT produced concording results with GNP-MB and GNP-TB combination rendering only approx. 80% biofilm inhibition (both developing and mature) in contrast to solitary effects of these complexes which induced approx. 40%–50% viable mature as well as immature biofilm at a concentration of 100 μg/ml of respective formulations. Since *C*. *albicans* has been suggested to establish itself in the form of biofilms on host tissues (preferably skin and mucosa) [40–42]. The hyphae present in the biofilm help fungal cells to penetrate deeper into tissues to establish infection, these results corroborate that GNP-MB and GNP-TB combination can play a significant role in depleting *C*. *albicans* biofilm on medical implants and host tissues. To evaluate effect of PDT on *C*. *albicans* morphognesis, we determined expression level of two hypha specific genes viz. *ALS3* and *HYR1* under hypha inducing conditions. While *ALS3* (agglutination like sequence 3) is a cell wall protein [43], *HYR1* encodes a glycosylphosphatidyl inositol—anchored protein that helps organism to avoid phagocyte mediated killing [44]. The transcript analysis study suggests that PDT causes down-regulation of both important genes (S2 Fig). Thus, seems to exert its antibiofilm effect via perturbation of cell wall integrity.

Besides antifungal potential against biofilms, the PDT employing GNP-MB and GNP-TB combination against yeast form of *C*. *albicans* resulted in pronounced cell killing (Fig 5C). The treatment with GNP conjugate combination was found to induce significant reduction of fungal load whereas treatment with single conjugate alone was found to be less effective than the combination. Interestingly, GNP-MB and GNP-TB combination was also found to be effective in killing *C*. *glabrata* as well (S3 Fig).

As *C*. *albicans* takes shelter inside macrophages during establishment phase, we ascertained if GNP-PS complex has relevance in treatment against intracellular C. *albicans*. It was observed that GNP-PS complex was efficiently engulfed by macrophages as exhibited by punctuate fluorescence (Fig 6). Intracellular abode of *C*. *albicans* not only makes them less susceptible to host antibody but also to available antibiotics. In such circumstances, photodynamic inactivation of *C*. *albicans* infection seems to have more relevance. The internalized GNP-complex was found to execute photodynamic inactivation of the fungal cells. In addition with the *in vitro* effect against *C*. *albicans* biofilms as well as on yeast cells, GNP-MB and GNP-TB combination was found to cause significant killing of fungal cells inside the macrophages (Fig 7A and 7B).

The GNP-PS conjugate combination (GNP-MB+GNP-TB) vs GNP-MB or vs GNP-TB solitary therapy clearly established potential of PDT against cutaneous *C*. *albicans* infection in mouse model (Figs 7C and 8). We assessed the potential of PDT in treatment of oral candidiasis as immuno-compromised subjects are equally susceptible to oral *C*. *albicans* infection [5, 45, 46]. The treatment with GNP-MB and GNP-TB was successful in significantly reducing the yeast cells and hyphal filaments penetrating the papillae in tongue dorsum of mice (Fig 9). Previous reports have also suggested PDT to be an effective strategy for treatment of oral *C*. *albicans* infection [47–50]. The tissue (skin and tongue) penetrating hyphal filaments can be significantly inhibited by PDT employing GNP-MB and GNP-TB combination based PDT. It should be conceived that the mouse models infected with such higher densities of fungi are somewhat unnatural as clinical skin wounds and oral cavity harbour a far lesser number of fungal pathogen. We anticipate better efficacy of the PDT employing combination GNP-PS conjugates against *C*. *albicans* topical skin and oral infections in clinical settings.

Finally, we reckon that the dual PS conjugated GNPs based PDT offers a novel system to suppress both topical as well as oral candidiasis in model animals. The strategy may have clinical relevance if combined with chemotherapy for successful treatment of candidiasis.

## Conclusions

The present study highlights potential of GNP-MB and GNP-TB combination based therapy in downsizing both biofilm as well as planktonic *C*. *albicans* population. The efficiency of GNP conjugates based PDT was further established by its ability to suppress superficial skin as well as oral *C*. *albicans* infection in BALB/c mice. Consequently, the data of the present study suggest that photodynamic effect of the GNP conjugate mixture can have potential application in treatment of cutaneous *C*. *albicans* infection and can be extended to inhibit the fungal biofilms on medical implants and catheters as well.

## Supporting Information

S1 Fig The original or un-normalized ultraviolet-visible absorption spectra of gold nanoparticle photosensitizer conjugate. **(A)** MB conjugation to GNP leads to appearance of a small peak at 540 nm, intrinsic feature of GNP, which was absent in pure MB spectrum **(B)** GNP-TB conjugate showing additional peak at 540 nm **(C)** Spectrum obtained upon mixing GNP-MB and GNP-TB conjugates.
(TIF)

S2 Fig Expression of hyphae inducing genes in *C*. *albicans* biofilm. Different expression of genes *ALS3* and *HYR1* following the treatment with various GNP-PS preparations. *ACT1* was taken as internal control. Data are means of three determinants ±SD and represent three replicates. GNP-MB+GNP-TB vs Control, *P*<0.005.
(TIF)

S3 Fig *In vitro* photodynamic inactivation of *C*. *glabrata*. Various GNP-PS preparations were incubated with *C*. *glabrata* yeast cells and then exposed to respective light source. The irradiated cell suspensions (50 μl) were plated onto YPD agar plates for counting CFU. Data are means of three determinants ±SD and represent three replicates. GNP-MB+GNP-TB vs Control, *P*<0.005.
(TIF)

S1 Protocol Photodynamic inactivation of *C*. *glabrata* cells *in vitro*. GNP-PS conjugate based photodynamic inactivation of planktonic *C*. *glabrata* cells was also performed.
(DOCX)

S2 Protocol Gene expression in *C*. *albicans* biofilm. The mRNA abundances of the genes of interest were quantitated in biofilms by RT-PCR.
(DOCX)
