# Supplementary figures and images for: Gold Nanoparticle-Photosensitizer Conjugate Based Photodynamic Inactivation of Biofilm Producing Cells: Potential for Treatment of C. albicans Infection in BALB/c Mice
Source: PLoS One. 2015 Jul 6;10(7):e0131684. doi: 10.1371/journal.pone.0131684 (PMC4493101; doi:10.1371/journal.pone.0131684)

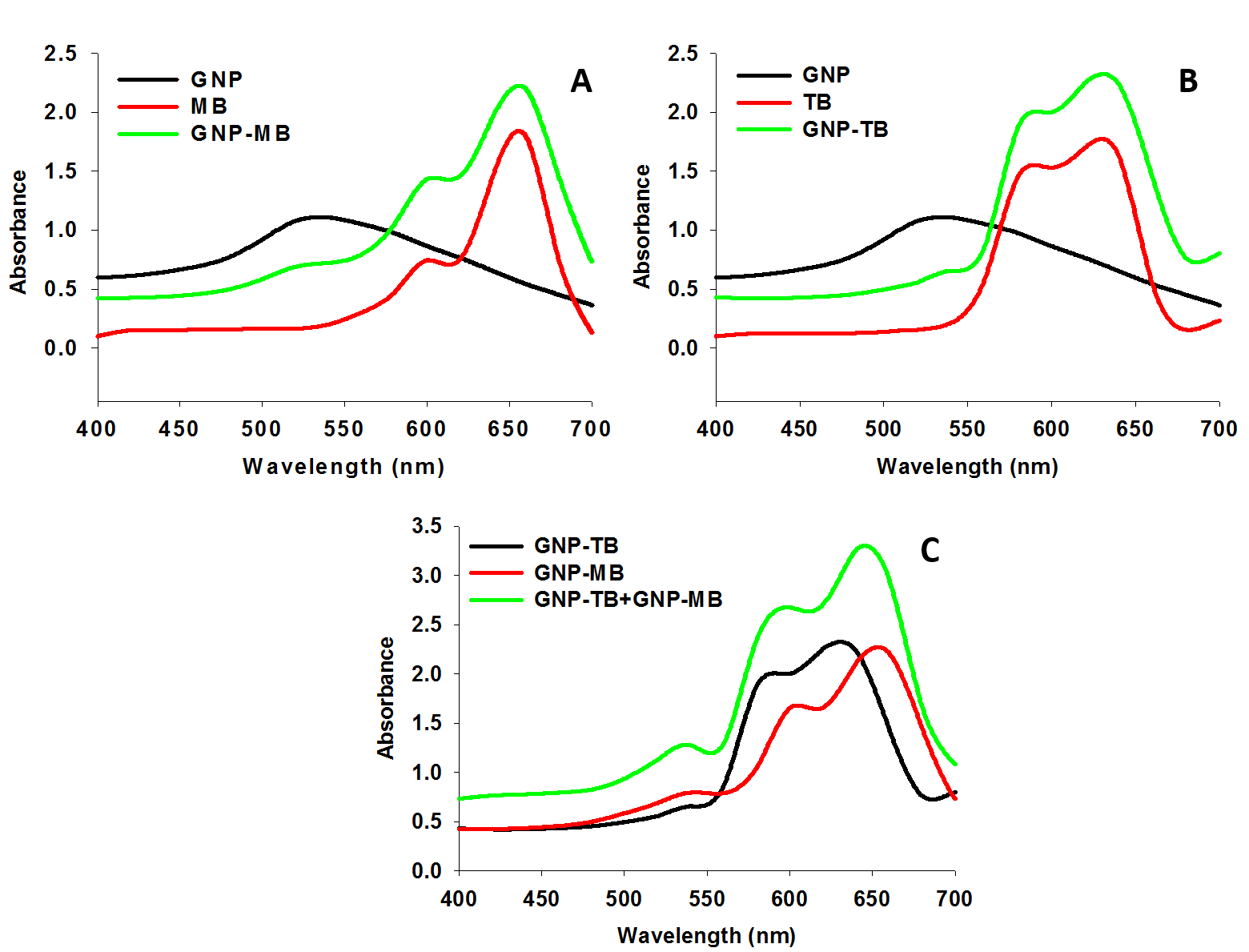

Supplement: S1 Fig — (A) MB conjugation to GNP leads to appearance of a small peak at 540 nm, intrinsic feature of GNP, which was absent in pure MB spectrum (B) GNP-TB conjugate showing additional peak at 540 nm (C) Spectrum obtained upon mixing GNP-MB and GNP-TB conjugates. (TIF) [file pone.0131684.s001.tif]

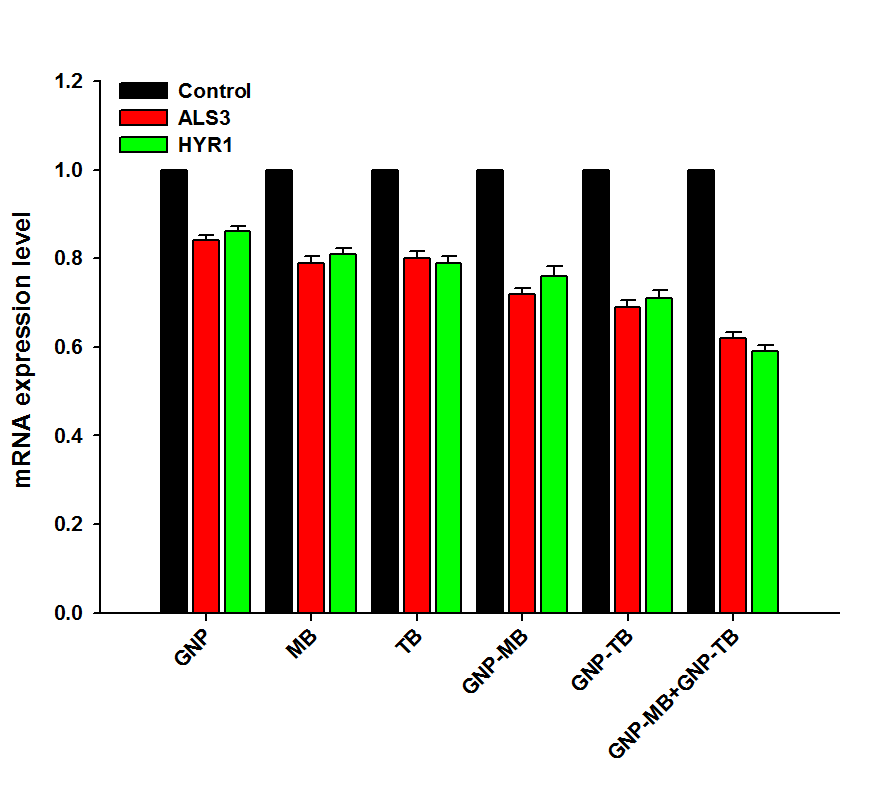

Supplement: S2 Fig — Different expression of genes ALS3 and HYR1 following the treatment with various GNP-PS preparations. ACT1 was taken as internal control. Data are means of three determinants ±SD and represent three replicates. GNP-MB+GNP-TB vs Control, P<0.005. (TIF) [file pone.0131684.s002.tif]

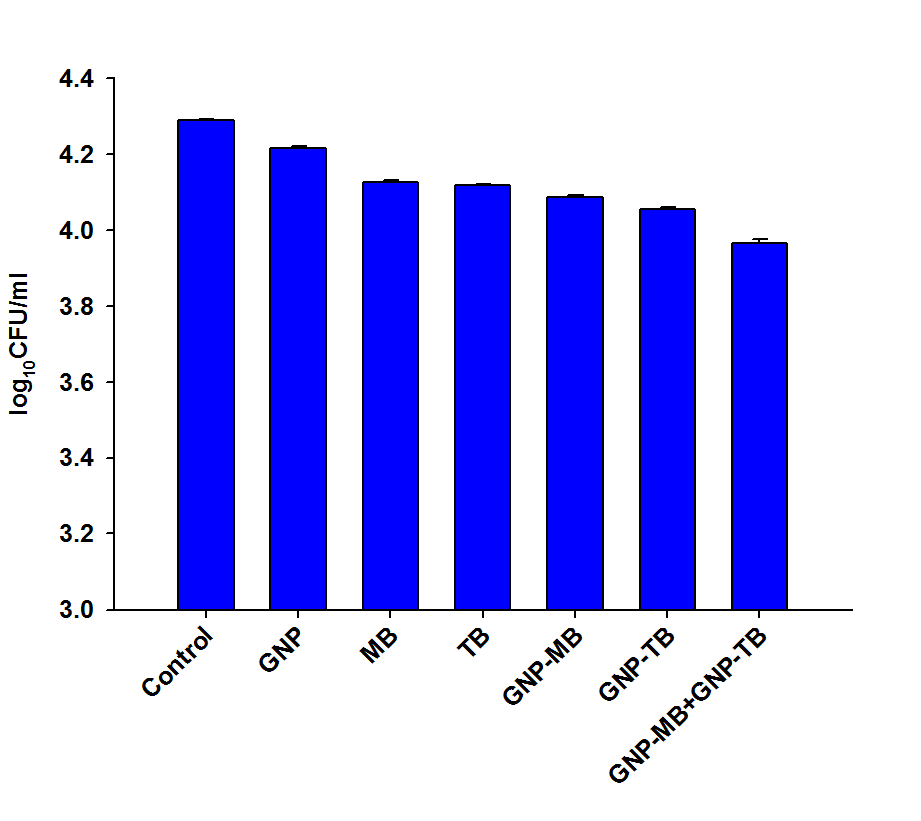

Supplement: S3 Fig — Various GNP-PS preparations were incubated with C. glabrata yeast cells and then exposed to respective light source. The irradiated cell suspensions (50 μl) were plated onto YPD agar plates for counting CFU. Data are means of three determinants ±SD and represent three replicates. GNP-MB+GNP-TB vs Control, P<0.005. (TIF) [file pone.0131684.s003.tif]
