# Supplementary material for: Gold Nanoparticle-Photosensitizer Conjugate Based Photodynamic Inactivation of Biofilm Producing Cells: Potential for Treatment of C. albicans Infection in BALB/c Mice
Source: PLoS One. 2015 Jul 6;10(7):e0131684. doi: 10.1371/journal.pone.0131684 (PMC4493101; doi:10.1371/journal.pone.0131684)
Supplement: S1 Protocol — GNP-PS conjugate based photodynamic inactivation of planktonic C. glabrata cells was also performed. (DOCX) [file pone.0131684.s004.docx]

Photodynamic therapy was also performed on *C. glabrata* cells. The cell suspension at the density of 10^7^ CFU/ml in PBS was dispensed into a six well culture plate and incubated with 200 µg/ml (50 µl) of various formulations (naked GNP, GNP-MB, GNP-TB and GNP-MB+GNP-TB) for 30 min at room temperature in the dark. Further, the cells were irradiated with the light source employing respective filters of 662nm and 635nm. During illumination, the lids of the culture plates were removed. Aliquots of 50 µl of the fungal suspension were withdrawn and plated onto the YPD agar plates to determine residual fungal cells.
