# Supplementary material for: Gold Nanoparticle-Photosensitizer Conjugate Based Photodynamic Inactivation of Biofilm Producing Cells: Potential for Treatment of C. albicans Infection in BALB/c Mice
Source: PLoS One. 2015 Jul 6;10(7):e0131684. doi: 10.1371/journal.pone.0131684 (PMC4493101; doi:10.1371/journal.pone.0131684)
Supplement: S2 Protocol — The mRNA abundances of the genes of interest were quantitated in biofilms by RT-PCR. (DOCX) [file pone.0131684.s005.docx]

Quantitative real-time reverse transcription-PCR (RT-PCR) was used to compare mRNA abundances of the genes of interest. Biofilms grown on plastic coverslips were exposed to various GNP-PS formulations and treated with PDT as described in Materials and Methods section of the main body of manuscript. The biofilm material left after treatment was scraped off from the surface of coverslips, re-suspended in PBS and centrifuged to obtain a pellet containing biofilm matrix. Total RNA was extracted from biofilm following the procedure published elsewhere [1]. The TaqMan MGB probe and primer sets were designed for the target genes ALS3 and HYR1, using Primer Express 1.5 software (Applied Biosystems, Foster City, Calif.) [2]. ACT1 was used as internal control. Primer sequences used for amplification of the genes are as follows:

ALS3f: 5’- CCACTTCACAATCCCCATC -3’;

ALS3r: 5’-CAGCAGTAGTAGTAACAGTAGTAGTTTCATC-3’;

HYR1f: 5’TTGTTTGCGTCATCAAGACTTTG-3’;

HYR1r: 5’GTCTTCATCAGCAGTAACACAACCA-3’;

ACT1f : 5’-AGCTTTGTTCAGACCAGCTGATT-3’;

ACT1r: 5’-TTGACCAAACCACTTTCAACTCC-3’.

The quantitative data analysis was completed using an Applied Biosystem Real Time PCR System. The comparative expression value was calculated using the following formula: fold change =2^-ΔΔCt^ [3].

**References**

1. **Chandra J, Kuhn DM, Mukherjee PK, Hoyer LL, McCormick T, et al.** (2001) Biofilm formation by the fungal pathogen Candida albicans: development, architecture, and drug resistance. J Bacteriol. 183: 5385-94.

# 2. [Green CB](http://www.ncbi.nlm.nih.gov/pubmed?term=Green%20CB%5BAuthor%5D&cauthor=true&cauthor_uid=14766904), [Cheng G](http://www.ncbi.nlm.nih.gov/pubmed?term=Cheng%20G%5BAuthor%5D&cauthor=true&cauthor_uid=14766904), [Chandra J](http://www.ncbi.nlm.nih.gov/pubmed?term=Chandra%20J%5BAuthor%5D&cauthor=true&cauthor_uid=14766904), [Mukherjee P](http://www.ncbi.nlm.nih.gov/pubmed?term=Mukherjee%20P%5BAuthor%5D&cauthor=true&cauthor_uid=14766904), [Ghannoum MA](http://www.ncbi.nlm.nih.gov/pubmed?term=Ghannoum%20MA%5BAuthor%5D&cauthor=true&cauthor_uid=14766904) et al. (2004) RT-PCR detection of Candida albicans ALS gene expression in the reconstituted human epithelium (RHE) model of oral candidiasis and in model biofilms. [Microbiology.](http://www.ncbi.nlm.nih.gov/pubmed/14766904) 150(Pt 2): 267-75.

3. **Li Y, Ma Y, Zhang L, Guo F, Ren L, Yang R, Li Y, Lou H** (2012) In vivo inhibitory effect on the biofilm formation of Candida albicans by liverwort derived riccardin D. PLoS One. 7: e35543.
